# Extent of lymphadenectomy dictates adjuvant chemotherapy benefit in primarily resected esophageal squamous cell carcinoma: a multi-cohort validation

**DOI:** 10.3389/fonc.2026.1920549

**Published:** 2026-10-29

**Authors:** Yicheng Huang, Xuanhao Lin, Yanhong Lin, Ziyang Han

**Affiliations:** 1 Department of Thoracic Surgery, Fujian Medical University Union Hospital, Fuzhou, Fujian, China; 2 Key Laboratory of Cardio-Thoracic Surgery (Fujian Medical University), Fuzhou, Fujian, China; 3 Key Laboratory of Gastrointestinal Cancer (Fujian Medical University), Ministry of Education, Fuzhou, Fujian, China; 4 Fujian Key Laboratory of Tumor Microbiology, Department of Medical Microbiology, Fujian Medical University, Fuzhou, Fujian, China; 5 Clinical Research Center for Thoracic Tumors of Fujian Province, Fujian Medical University, Fuzhou, Fujian, China

**Keywords:** adjuvant chemotherapy, esophageal squamous cell carcinoma, lymph node dissection, nomogram, prognostic model, propensity score matching, therapeutic de-escalation

## Abstract

**Background:**

While primary surgery remains a crucial treatment for resectable esophageal squamous cell carcinoma (ESCC), the uniform application of postoperative adjuvant chemotherapy for pathologically node-positive patients exposes many to severe systemic toxicity. This study hypothesizes the existence of a specific lymph node dissection (LND) threshold that stratifies the survival benefit of adjuvant chemotherapy and aims to define this cutpoint to enable safe therapeutic de-escalation.

**Methods:**

We conducted a multi-cohort retrospective study using data from the Surveillance, Epidemiology, and End Results (SEER) database (n = 753, training cohort) and a Chinese single-center repository (n = 447, validation cohort) of patients with ESCC who underwent primary radical esophagectomy without radiotherapy. The optimal prognostic cutpoint for LND was mathematically determined using X-tile software. An interactive Cox proportional hazards nomogram incorporating an LND-by-chemotherapy interaction term was developed and validated. Subsequently, layer-specific 1:1 propensity score matching (PSM) was performed within separate surgical strata of the validation cohort to assess chemotherapy efficacy.

**Results:**

X-tile analysis established 21 lymph nodes as the optimal LND threshold. Multivariable analysis confirmed that adequate LND (≥ 21 nodes) and adjuvant chemotherapy were independent protective prognostic factors. The interactive nomogram demonstrated good discrimination and calibration across both populations. Crucially, layer-specific PSM in the external validation cohort revealed divergent therapeutic responses dependent on surgical quality. For patients with suboptimal LND (< 21 nodes), adjuvant chemotherapy provided a significant survival benefit over surgery alone (P = 0.048). In contrast, for patients achieving an adequate LND (≥21 nodes), the addition of adjuvant chemotherapy yielded no further survival advantage (P = 0.085).

**Conclusions:**

Achieving the removal of ≥ 21 lymph nodes serves as a robust anatomical biomarker that fundamentally alters the postoperative therapeutic landscape for ESCC. Patients with suboptimal LND require adjuvant chemotherapy to compensate for potential residual disease, whereas those achieving the 21-node clearance threshold derive no additional survival benefit from systemic interventions and can be safely exempted from cytotoxic over-treatment.

## 1 Introduction

According to the GLOBOCAN global cancer statistics, esophageal cancer remains a highly aggressive malignancy worldwide, ranking as the seventh leading cause of cancer-related mortality and accounting for over 440,000 deaths annually (1). Esophageal squamous cell carcinoma (ESCC) comprises approximately 90% of all cases and represents the predominant histological subtype (2, 3). Characterized by highly invasive behavior and an extensive multidirectional submucosal lymphatic network, ESCC typically carries a poor prognosis. Radical esophagectomy combined with systemic lymph node dissection (LND) remains the cornerstone of curative-intent treatment for resectable ESCC. Although neoadjuvant chemoradiotherapy combined with immunotherapy has been widely endorsed for locally advanced disease (4), upfront surgery still accounts for a substantial proportion of treatments in real-world practice. This phenomenon is predominantly driven by the inherent diagnostic limitations and high false-negative rates of preoperative staging modalities, such as endoscopic ultrasound and PET-CT, in detecting occult regional nodal micrometastases. Consequently, a substantial proportion of patients initially evaluated as clinically node-negative (cN0) undergo primary radical resection but are unexpectedly upstaged postoperatively to node-positive (pN+) disease (5, 6).

For this specific high-risk subpopulation that has missed the therapeutic window for neoadjuvant intervention, postoperative adjuvant chemotherapy serves as the standard therapeutic modality. However, the uniform application of adjuvant therapy—dictated solely by static anatomical TNM classifications (i.e., pN+ status)—inevitably exposes a broad spectrum of patients to severe systemic toxicities and a deterioration in quality of life (6, 7). The thoroughness of LND not only is essential for accurate pathological staging to mitigate stage migration but also acts as a direct therapeutic intervention by mechanically eliminating the regional minimal residual disease (MRD) burden (8–12). While the National Comprehensive Cancer Network (NCCN) guidelines recommend a minimum harvest of 15 lymph nodes to ensure basic staging adequacy (5), current clinical paradigms generally overlook the moderating interaction effect between surgical clearance quality and the absolute efficacy of subsequent systemic interventions (13, 14). To date, a crucial knowledge gap persists: it remains unknown whether a quality-controlled, extended lymphadenectomy can achieve absolute regional micrometastatic clearance, thereby justifying therapeutic de-escalation via the safe exemption from adjuvant chemotherapy in a highly selected subpopulation (15–18). To address this crucial clinical dilemma, we hypothesized the existence of a definitive surgical threshold of LND thoroughness that stratifies the survival benefit derived from postoperative adjuvant chemotherapy. The primary objective of this study was to define this optimal LND cutpoint and subsequently evaluate the safety of exemption from chemotherapy within the optimal surgical quality stratum.

The significance of this study lies in its methodological approach to addressing the mathematical conflicts inherent in traditional linear prognostic models. By integrating product interaction terms and employing rigorous layer-specific propensity score matching (PSM) across distinct surgical strata, this study provides an empirically robust, cross-population-validated framework. Theoretically, it elucidates the biological interaction between regional macroscopic surgical clearance and systemic interventions. In practice, it yields an objective, quantitative prognostic nomogram that enables surgical oncologists to confidently implement personalized therapeutic de-escalation, thereby preventing cytotoxic overtreatment for adequately resected patients.

## 2 Materials and methods

### 2.1 Data sources and patient population

This study used data derived from two independent geographic cohorts: the population-based Surveillance, Epidemiology, and End Results (SEER) database (representing the Western training cohort) and a real-world repository from a high-volume Chinese single-center institution (representing the Eastern validation cohort). The data screening process designed to minimize potential confounding is illustrated in **Figure 1**. The inclusion criteria were restricted to patients with pathologically confirmed esophageal squamous cell carcinoma (ESCC) (2, 3) who underwent primary radical esophagectomy combined with systemic lymph node dissection (LND). To eliminate the profound therapeutic confounding of multimodality radiation and minimize age-related baseline frailty, patients who received any preoperative or postoperative radiotherapy were completely excluded (Has Rad = 0), and the upper age limit was truncated at 72 years (Age ≤ 72). Patients with missing follow-up data, an overall survival (OS) of 0 months, or incomplete pathological TNM staging were omitted. Ultimately, 753 patients from the SEER database were allocated to the model development (training) cohort, and 447 patients from the institutional real-world repository were included in the external validation cohort.

**Figure 1:**
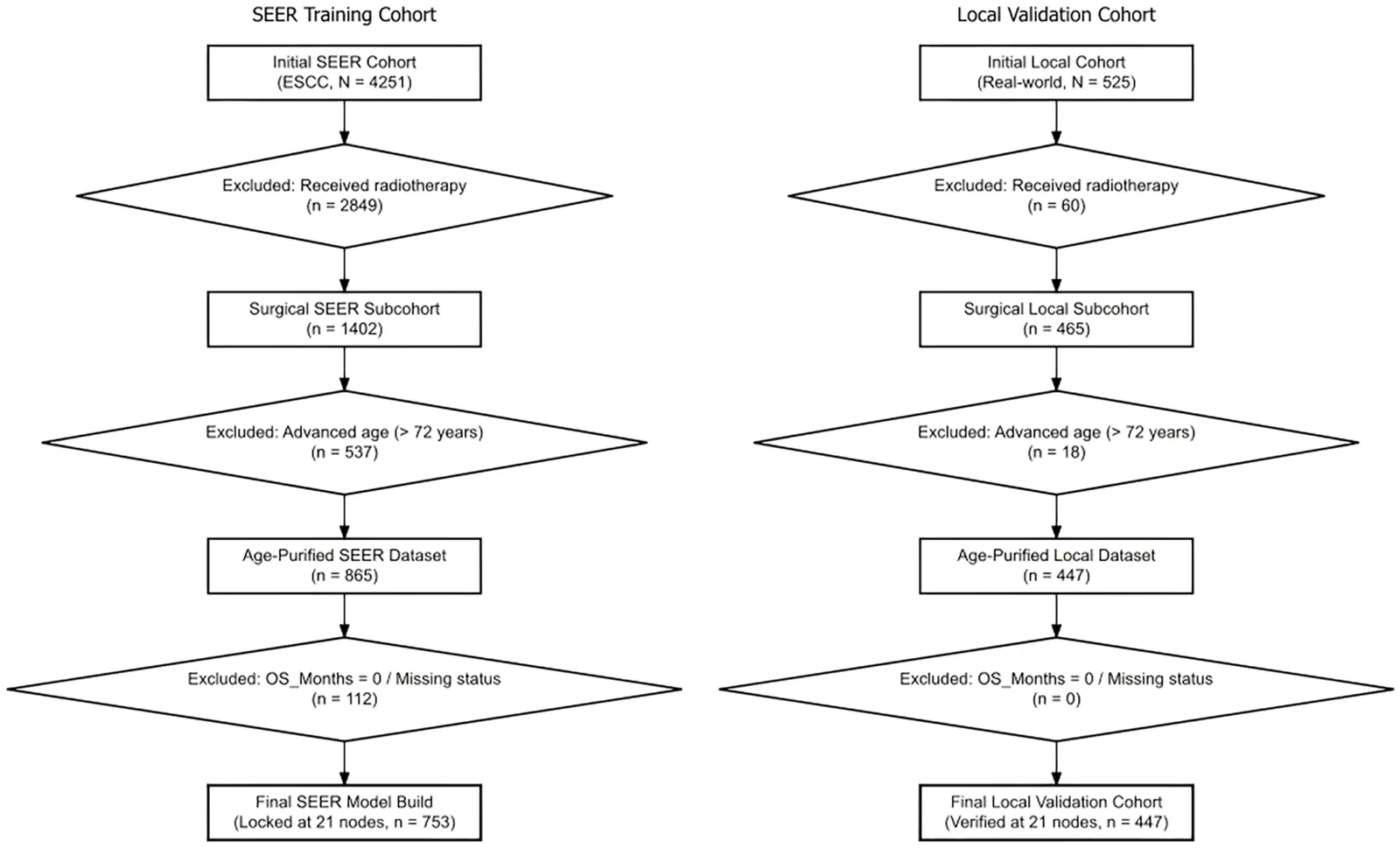
Flowchart of patient selection and multi-cohort screening workflow. ESCC, esophageal squamous cell carcinoma; LND, lymph node dissection; SEER, Surveillance, Epidemiology, and End Results.

### 2.2 Identification of the optimal lymph node dissection cutpoint

The extent of LND was quantified as a continuous variable representing the total number of regional lymph nodes harvested from the surgical specimen. To avoid arbitrary categorization or researcher bias common in clinical models, the optimal prognostic cutpoint for LND thoroughness was mathematically determined using data from the SEER training cohort and X-tile software (version 3.6.1, Yale University). The maximum log-rank chi-square statistic was computed along the continuous spectrum of node counts to define a threshold that maximized the long-term survival divergence between the suboptimal and adequate surgical quality groups.

### 2.3 Statistical analysis and interactive model construction

Baseline clinical characteristics of the Western training and Eastern validation cohorts were compared using Student’s t-test for continuous variables and Pearson’s chi-square test for categorical variables. Survival curves were estimated using the Kaplan-Meier method and systematically compared via the log-rank test. Hazard ratios (HRs) and their corresponding 95% confidence intervals (CIs) were estimated using univariable and multivariable Cox proportional hazards models.

An interactive prognostic nomogram was constructed by integrating a product interaction term (LND group × adjuvant chemotherapy) into the multivariable Cox regression model in the SEER cohort. This mathematical integration allows the nomogram to dynamically adjust the risk points assigned to chemotherapy status according to the specific surgical clearance stratum. Discrimination and calibration of the model were assessed using time-dependent receiver operating characteristic (ROC) curves and cross-population calibration plots, respectively.

### 2.4 Layer-specific propensity score matching

To validate the chemotherapy exemption hypothesis while minimizing indication bias (the clinical tendency to preferentially prescribe chemotherapy to advanced-stage or high-risk patients), an independent 1:1 nearest-neighbor propensity score matching (PSM) was performed within the separate surgical strata of the Chinese validation cohort. Patients were matched on pathological T stage (pT), pathological N stage (pN), age, and sex, using a caliper width of 0.25 to achieve optimal baseline balance within each layer. All statistical analyses were conducted using R software (version 4.3.2), and two-sided P-values < 0.05 were considered statistically significant.

## 3 Results

### 3.1 Baseline characteristics and optimal LND cutpoint selection

A comprehensive overview of the age-adjusted baseline characteristics for both cohorts is detailed in **Table 1**. Significant geographic disparities in surgical quality were observed: the Western SEER cohort exhibited a high proportion of suboptimal clearance, with 89.4% (n = 673) of patients undergoing removal of fewer than 21 nodes. Conversely, 83.2% (n = 372) of patients in the local Chinese cohort achieved extensive clearance. As demonstrated in **Figure 2**, X-tile analysis established the optimal prognostic threshold for LND thoroughness at 21 lymph nodes (log-rank chi-square = 41.42, P < 0.001). Consequently, patients were classified into either the suboptimal LND group (< 21 nodes) or the adequate LND group (≥ 21 nodes).

**Table 1:** Baseline clinicopathological characteristics of the SEER training cohort and the local validation cohort.

Characteristic	SEER cohort	Local cohort	P-value
Total patients	753	447	
Age (years, mean ± SD)	61.3 ± 7.5	58.8 ± 7.1	<0.0001
Sex			
Female	265 (35.2%)	111 (24.8%)	<0.0001
Male	488 (64.8%)	336 (75.2%)	
Pathological T stage			
T1	324 (43%)	133 (29.8%)	<0.0001
T2	77 (10.2%)	69 (15.4%)	
T3	171 (22.7%)	238 (53.2%)	
T4	181 (24%)	7 (1.6%)	
Pathological N stage			
N0	421 (55.9%)	257 (57.5%)	<0.0001
N1	261 (34.7%)	105 (23.5%)	
N2	51 (6.8%)	65 (14.5%)	
N3	20 (2.7%)	20 (4.5%)	
LND count			
<21	673 (89.4%)	75 (16.8%)	<0.0001
>=21	80 (10.6%)	372 (83.2%)	
Adjuvant chemotherapy			
No	458 (60.8%)	217 (48.5%)	<0.0001
Yes	295 (39.2%)	230 (51.5%)	

**Figure 2:**
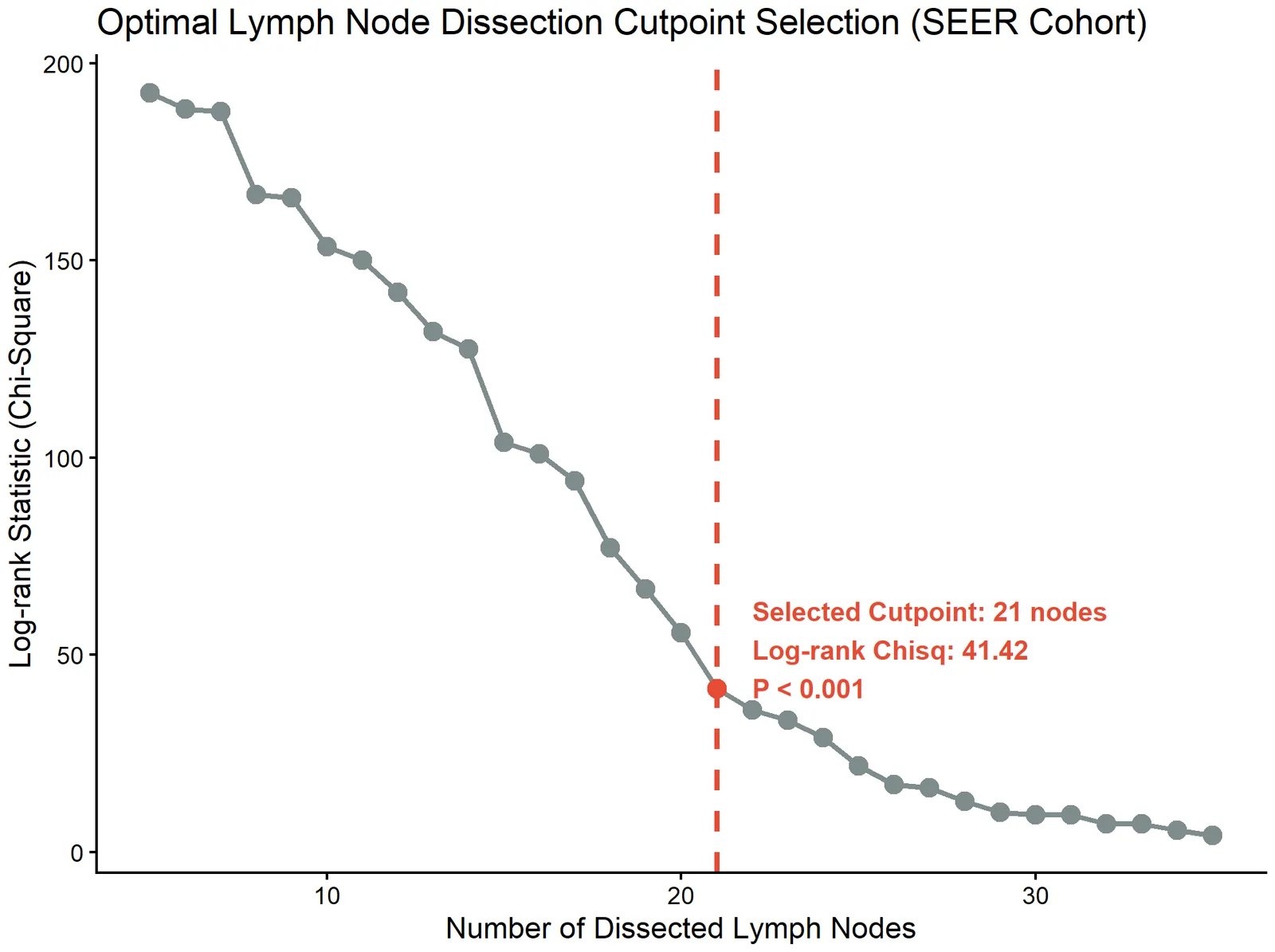
Determination of the optimal prognostic cutpoint for lymph node dissection (LND) count via X-tile software in the SEER cohort. LND, lymph node dissection; SEER, Surveillance, Epidemiology, and End Results.

### 3.2 Univariable and multivariable Cox proportional hazards analysis

In the SEER training cohort, the multivariable Cox regression model (**Table 2**) confirmed that adequate LND (≥ 21 nodes) emerged as an independent protective prognostic factor (HR = 0.56, 95% CI: 0.43-0.74, P < 0.001). Pathological pN1 (HR = 1.49, 95% CI: 1.24-1.79, P < 0.001), pN2 (HR = 1.55, 95% CI: 1.12-2.13, P = 0.0077), and pN3 (HR = 1.86, 95% CI: 1.15-3.00, P = 0.0113) stages, alongside pT4 stage (HR = 2.55, 95% CI: 2.05-3.18, P < 0.001), were significantly associated with poorer long-term survival. After accounting for surgical and staging covariates, adjuvant chemotherapy also exhibited an independent protective effect across the entire cohort (HR = 0.69, 95% CI: 0.58-0.82, P < 0.001). Kaplan-Meier survival analysis before and after 1:1 PSM for the LND threshold confirmed that adequate LND was associated with a significant survival advantage over suboptimal clearance in both the unmatched (**Figure 3A**, P < 0.0001) and matched (**Figure 3B**, P = 0.0015) subcohorts.

**Table 2:** Univariable and multivariable Cox proportional hazards regression analysis for overall survival in the SEER training cohort.

Variables	Univariable analysis		Multivariable analysis	
	HR (95% CI)	P-value	HR (95% CI)	P-value
Adjuvant chemotherapy				
No	Reference		Reference	
Yes	0.94 (0.81–1.1)	0.463	0.69 (0.58–0.82)	<0.001
Age (years)	1 (0.99–1.01)	0.632	1 (0.99–1.01)	0.449
Sex				
Female	Reference		Reference	
Male	1.42 (1.2–1.67)	<0.001	1.26 (1.07–1.49)	0.005
Pathological T stage				
T1	Reference		Reference	
T2	0.73 (0.55–0.97)	0.030	0.75 (0.56–1)	0.046
T3	1.19 (0.98–1.44)	0.087	1.1 (0.89–1.36)	0.376
T4	2.94 (2.41–3.59)	<0.001	2.55 (2.05–3.18)	<0.001
Pathological N stage				
N0	Reference		Reference	
N1	1.59 (1.35–1.88)	<0.001	1.49 (1.24–1.79)	<0.001
N2	1.58 (1.17–2.14)	0.003	1.55 (1.12–2.13)	0.008
N3	1.62 (1.03–2.54)	0.037	1.86 (1.15–3)	0.011
LND count				
< 21	Reference		Reference	
≥ 21	0.52 (0.4–0.68)	<0.001	0.56 (0.43–0.74)	<0.001

HR, hazard ratio; CI, confidence interval; SEER, Surveillance, Epidemiology, and End Results; LND, lymph node dissection.

The hazard ratios (HR) and 95% confidence intervals (CI) were estimated using univariable and multivariable Cox proportional hazards models. A P-value < 0.05 was considered statistically significant.

**Figure 3:**
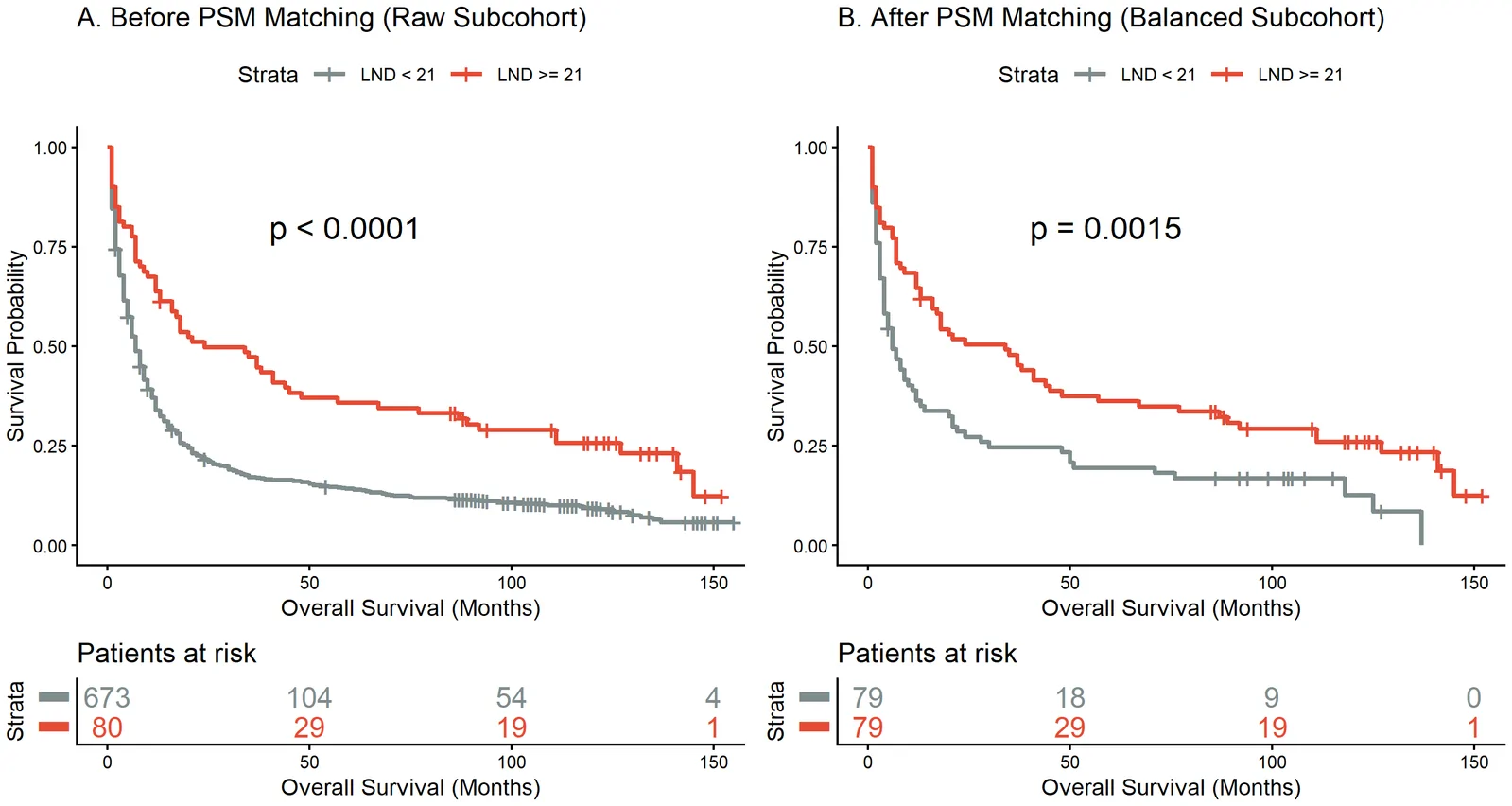
Kaplan-Meier overall survival curves stratified by the lymph node dissection (LND) threshold in the SEER cohort. **(A)** Survival curves before propensity score matching (PSM) and **(B)** survival curves after 1:1 PSM. LND, lymph node dissection; PSM, propensity score matching; SEER, Surveillance, Epidemiology, and End Results.

### 3.3 Development and evaluation of the interactive nomogram

To translate these findings into a precise clinical tool, an interactive prognostic nomogram was established (**Figure 4**). By incorporating the interaction term, the nomogram displays separate risk allocation axes. For patients in the suboptimal LND group (< 21 nodes), withholding chemotherapy (No) is associated with an increase of approximately 30 risk points. For patients in the adequate LND group (≥ 21 nodes), the risk points between the chemotherapy and surgery-only configurations converge completely, demonstrating therapeutic equivalence. The global model demonstrated robust discrimination, yielding 1-, 3-, and 5-year area under the curve (AUC) values of 0.735, 0.751, and 0.742 in the internal training cohort, respectively. Furthermore, in the external real-world validation cohort, the model maintained reliable predictive performance with 1-, 3-, and 5-year AUCs of 0.728, 0.663, and 0.642 (**Figure 5**). The 1-, 3-, and 5-year calibration curves demonstrated excellent agreement between the nomogram-predicted survival probabilities and the actual observations in both the SEER training and the cross-population external validation cohorts (**Figure 6**). Decision curve analysis (DCA) further substantiated the strong clinical utility of the interactive model, exhibiting a broad and robust net benefit profile. Specifically, the model provided a positive net benefit across a wide range of threshold probabilities, spanning up to approximately 80% in the training cohort and consistently up to a 50% risk threshold in the real-world validation cohort (**Figure 7**).

**Figure 4:**
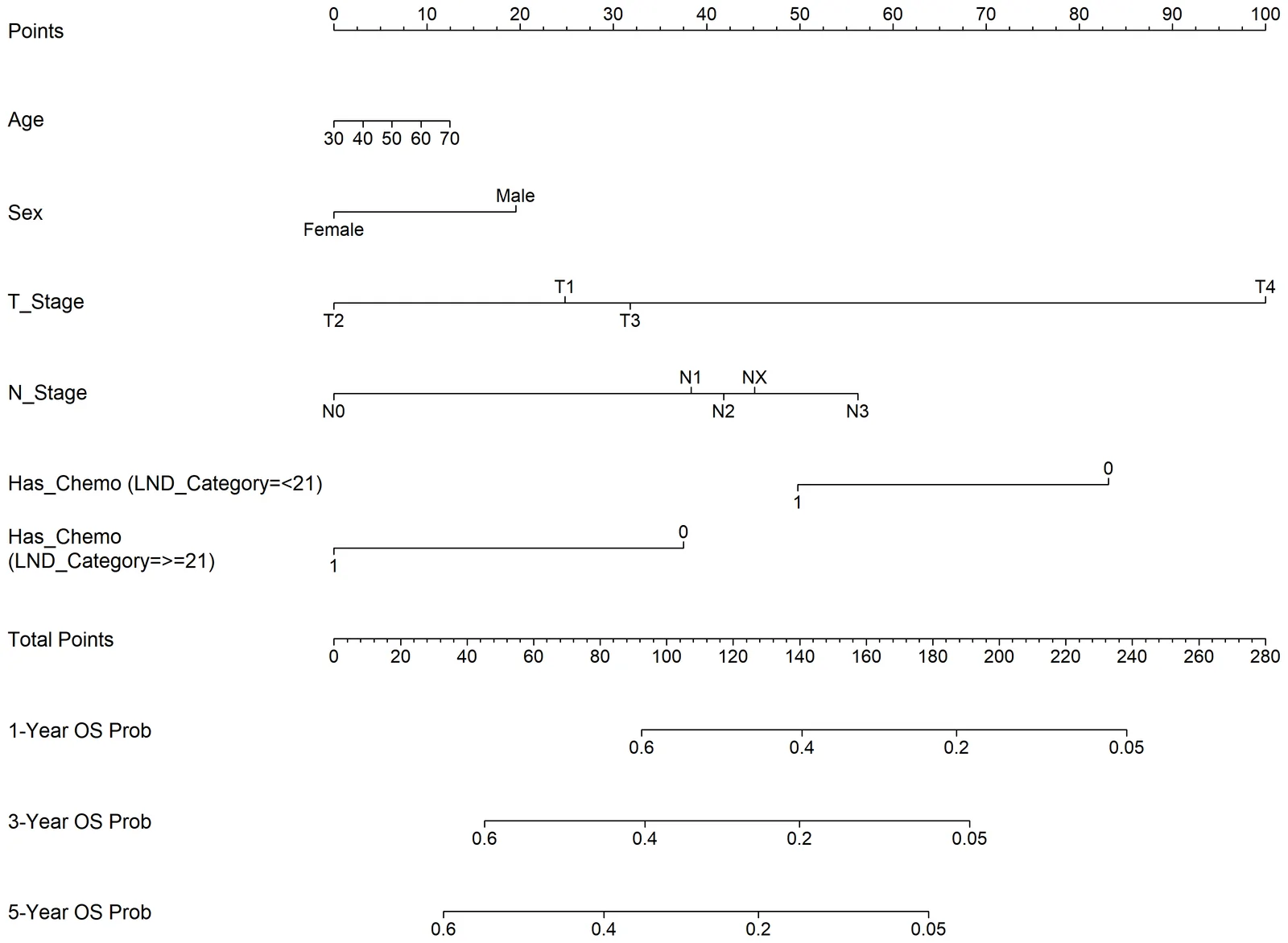
Development of an interactive prognostic nomogram for overall survival in the SEER training cohort. The nomogram integrates a product interaction term (LND Group × Adjuvant Chemo) to dynamically assign risk points based on specific surgical clearance strata. LND: Lymph Node Dissection; SEER: Surveillance, Epidemiology, and End Results.

**Figure 5:**
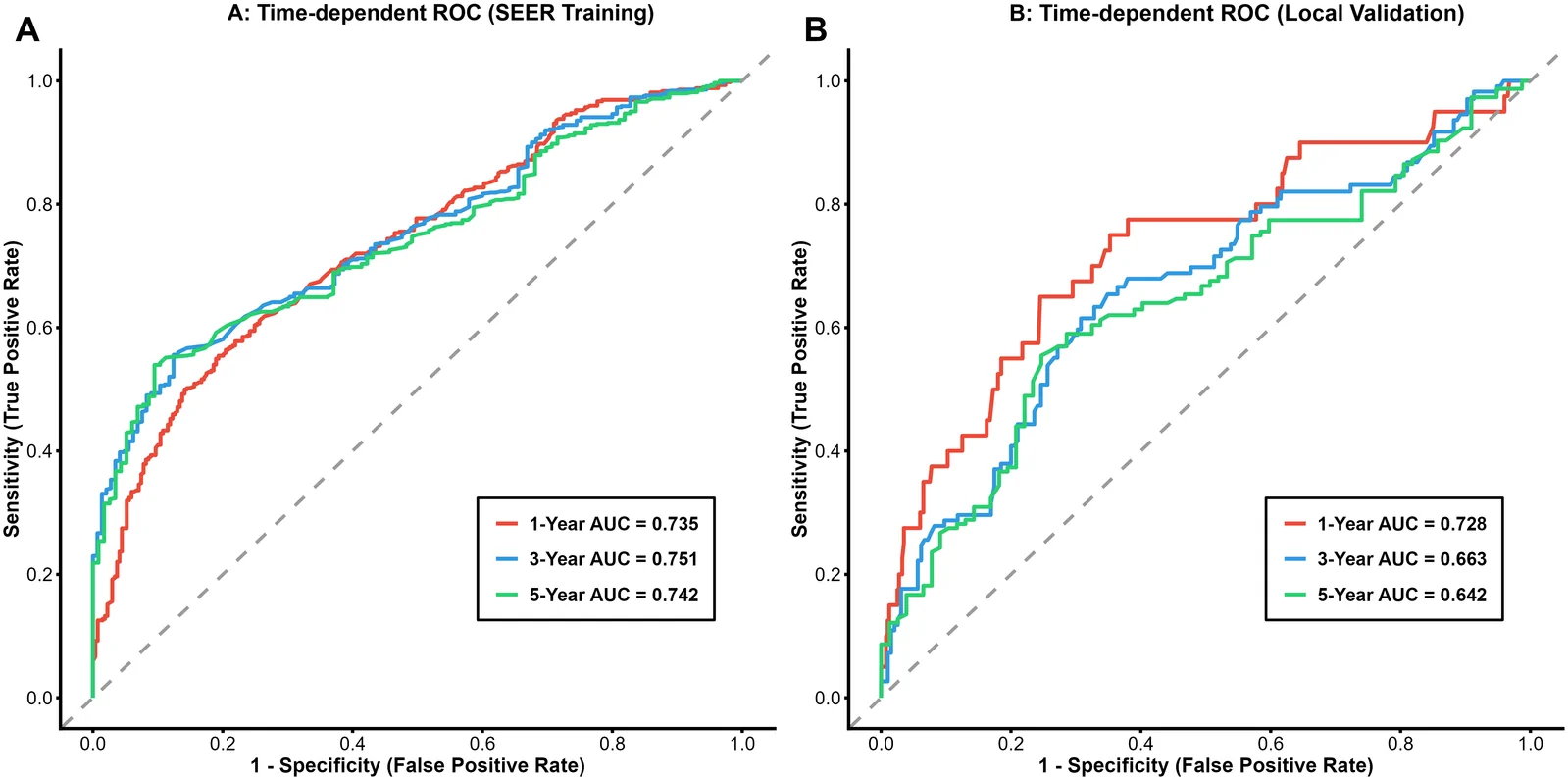
Time-dependent receiver operating characteristic (ROC) curves of the interactive nomogram for predicting overall survival. **(A)** 1-, 3-, and 5-year ROC curves in the SEER training cohort and **(B)** 1-, 3-, and 5-year ROC curves in the external validation cohort. ROC, receiver operating characteristic; SEER, Surveillance, Epidemiology, and End Results.

**Figure 6:**
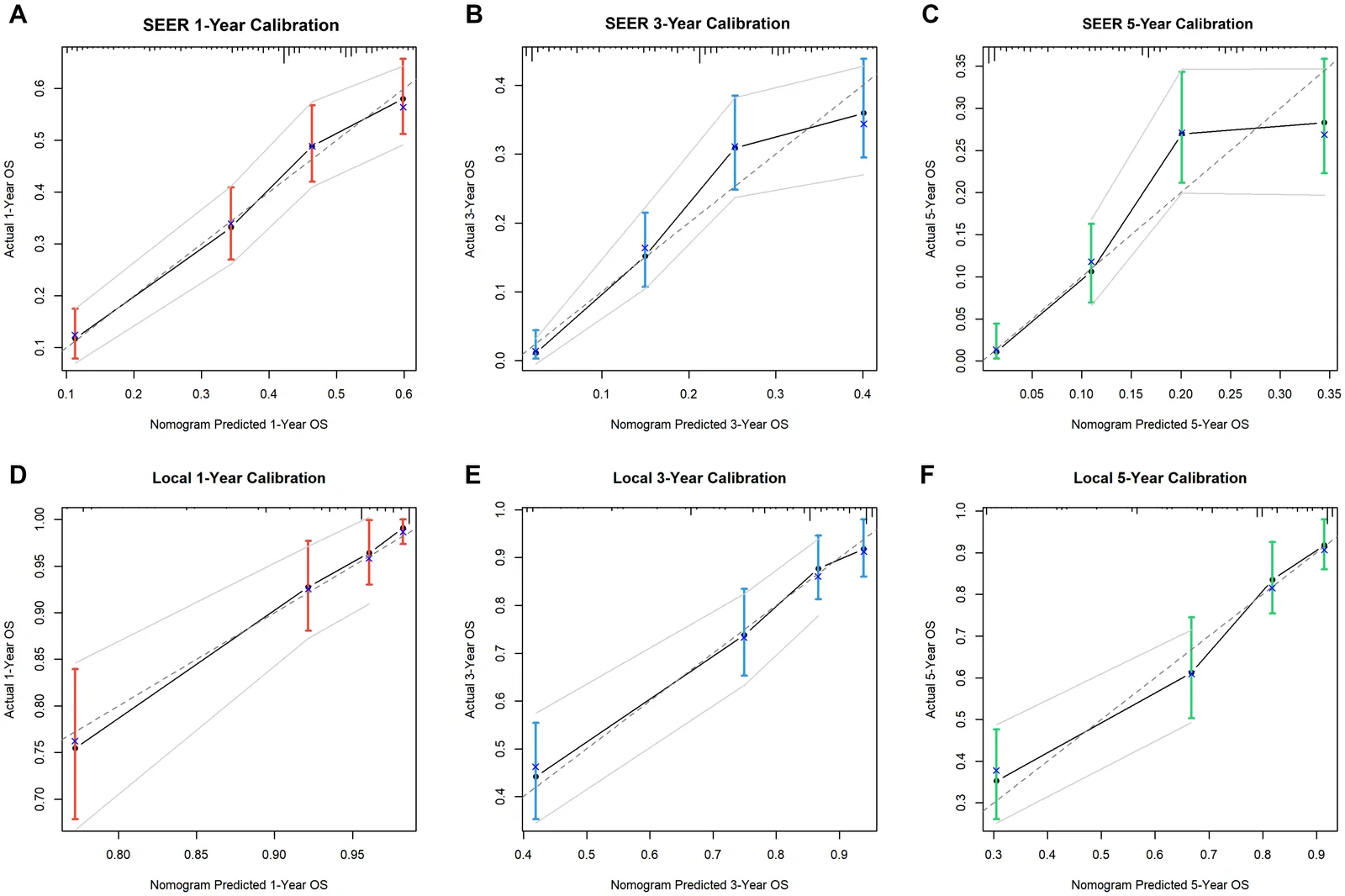
Calibration curves of the interactive nomogram for predicting overall survival at 1, 3, and 5 years. Panel **(A)** 1-year calibration in the SEER training cohort; Panel **(B)** 3-year calibration in the SEER training cohort; Panel **(C)** 5-year calibration in the SEER training cohort; Panel **(D)** 1-year calibration in the external real-world validation cohort; Panel **(E)** 3-year calibration in the external real-world validation cohort; and Panel **(F)** 5-year calibration in the external real-world validation cohort. The dashed line represents the performance of an ideal model, while the solid lines represent the observed performance of the proposed nomogram. Error bars indicate 95% confidence intervals. SEER: Surveillance, Epidemiology, and End Results.

**Figure 7:**
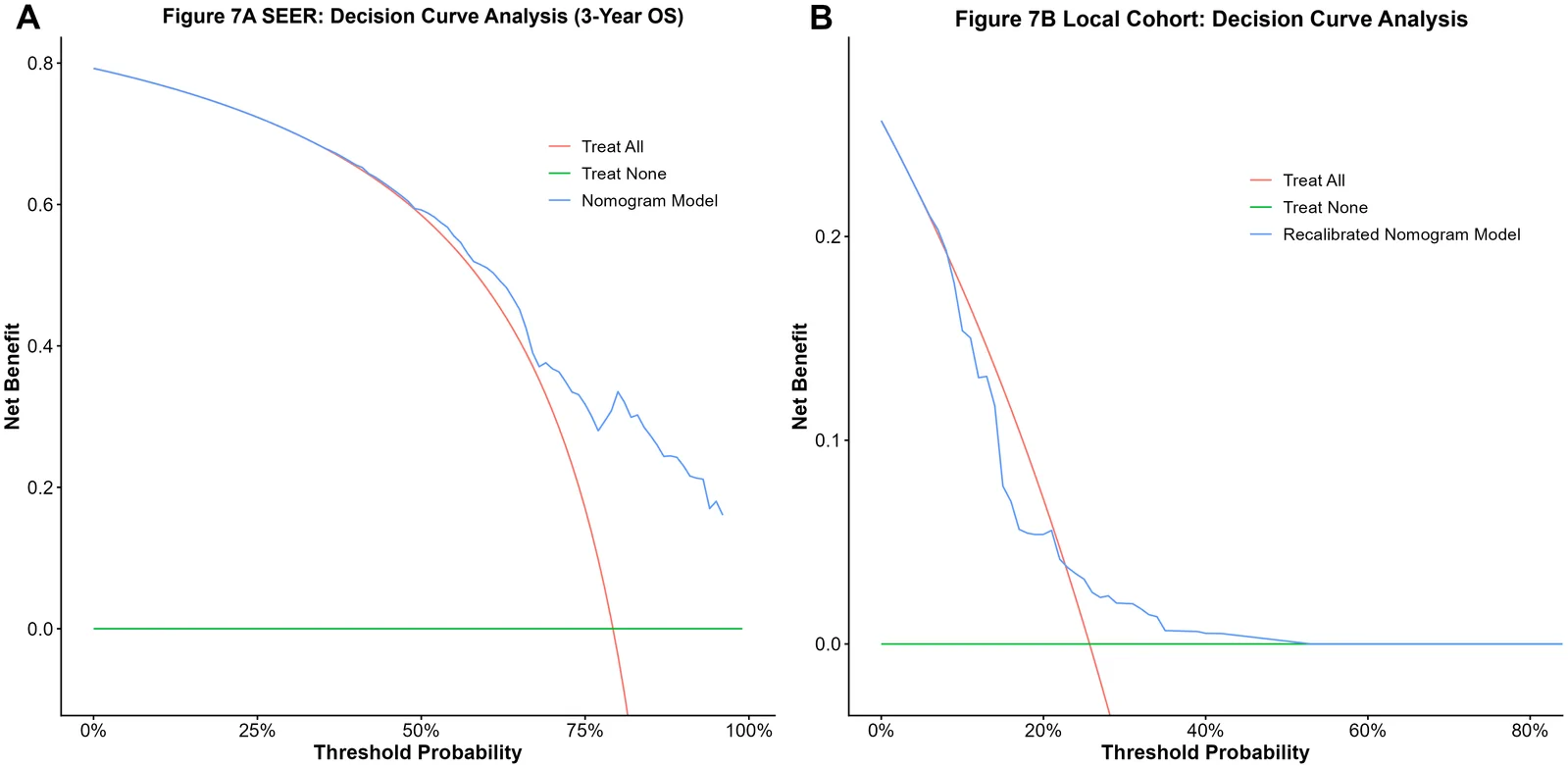
Decision curve analysis (DCA) of the interactive nomogram for predicting overall survival. **(A)** DCA curves in the SEER training cohort at 1, 3, and 5 years. **(B)** DCA curves in the external real-world validation cohort at 1, 3, and 5 years. The x-axis indicates the threshold probability, and the y-axis indicates the net benefit. SEER: Surveillance, Epidemiology, and End Results.

### 3.4 Global chemotherapy benefit vs. layer-specific PSM exemption validation

When evaluating adjuvant chemotherapy across the unstratified all-patient populations after global 1:1 PSM (**Figures 8A, B**), chemotherapy was associated with a significant survival detriment in the SEER cohort (P = 0.029), whereas a non-significant trend was observed in the Chinese local cohort (P = 0.077). To clarify this variance, an independent, layer-specific PSM was conducted within the distinct surgical strata of the Chinese validation cohort (**Figures 8C, D**). Within the suboptimal LND stratum (< 21 nodes; **Figure 8C**), where pathological stages were balanced (n = 21 vs. n = 21), the addition of adjuvant chemotherapy (red line) provided a significant survival benefit compared with surgery alone (P = 0.048). Within the quality-controlled, adequate LND stratum (≥ 21 nodes; **Figure 8D**), where 213 balanced patient pairs were compared (n = 213 vs. n = 213), the long-term survival curves of the adjuvant chemotherapy and surgery-only groups merged completely, yielding a non-significant difference (P = 0.085). This confirms that once radical clearance clears the regional tumor burden, further systemic chemotherapy provides no additional survival benefit, successfully achieving safe therapeutic de-escalation in the real world.

**Figure 8:**
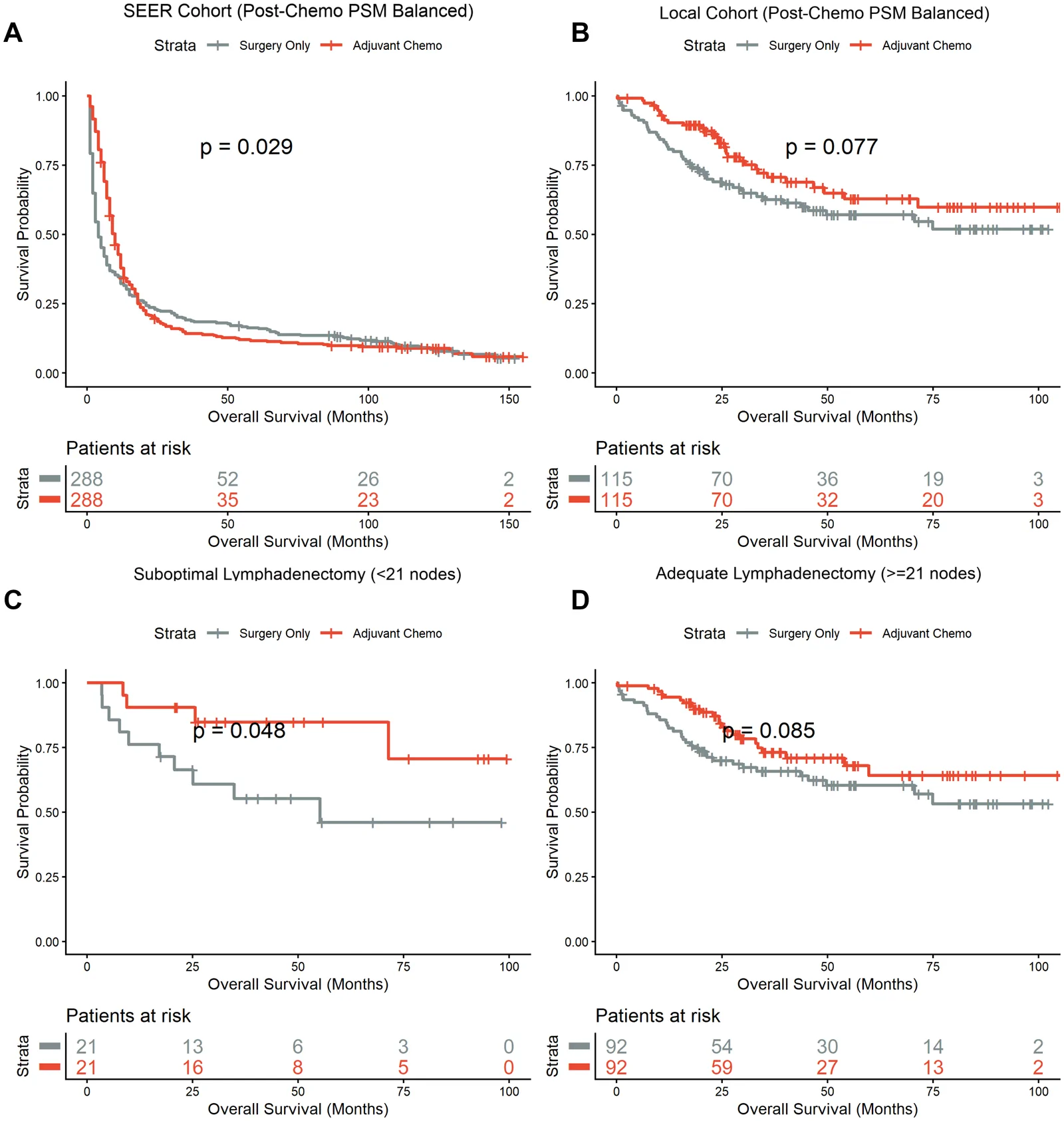
Layer-specific Kaplan-Meier analysis of overall survival to validate the efficacy of adjuvant chemotherapy across different lymph node dissection (LND) strata. **(A)** Survival curves for the global SEER cohort after 1:1 propensity score matching (PSM). **(B)** Survival curves for the global validation cohort after 1:1 PSM. **(C)** Survival curves within the suboptimal LND stratum (< 21 nodes) of the validation cohort after matching. **(D)** Survival curves within the adequate LND stratum (≥ 21 nodes) of the validation cohort after matching, demonstrating the therapeutic equivalence between surgery alone and surgery plus adjuvant chemotherapy. LND: lymph node dissection; PSM: propensity score matching; SEER: Surveillance, Epidemiology, and End Results.

## 4 Discussion

The clinical management of patients with esophageal squamous cell carcinoma (ESCC) who undergo primary surgical resection but experience unexpected pathological nodal upstaging (pN+) remains a subject of considerable debate (19, 20). While modern guidelines strongly recommend neoadjuvant treatment for locally advanced disease (4), upfront surgery remains common in clinical practice due to the high false-negative rates of endoscopic ultrasound and PET-CT staging. For these upstaged individuals, adjuvant chemotherapy has traditionally been recommended based solely on their pN+ status (21–24). The principal finding of this study is the empirical verification that surgical quality—specifically quantified by a threshold of 21 harvested lymph nodes—acts as a powerful moderator of adjuvant chemotherapy efficacy, allowing for the exemption from cytotoxic treatments in a highly selected subpopulation (25, 26).

The mathematical identification of the 21-node threshold in our study corresponds to the complex anatomical reality of esophageal lymphatic drainage. Due to its extensive multidirectional submucosal lymphatic network, ESCC frequently exhibits unpredictable “skip metastases,” particularly to high-risk sanctuary zones such as the recurrent laryngeal nerve (RLN) chains and subcarinal regions (15). While harvesting 15 nodes meets the minimum requirement for staging adequacy (5), achieving a yield of ≥ 21 nodes in clinical practice typically reflects the successful performance of a standardized, extended two-field or three-field lymphadenectomy. This scale of surgical thoroughness ensures the comprehensive clearance of occult micrometastatic niches that standard or suboptimal resections might omit. Consequently, the 21-node cutpoint is not merely a statistical artifact; rather, it serves as a robust anatomical proxy for achieving adequate regional disease control. From an oncological perspective, our stratified findings following independent propensity score matching (**Figure 8**) further elucidate the biological interaction between regional macroscopic clearance and systemic intervention. In patients who underwent a suboptimal lymphadenectomy (< 21 nodes), regional failure due to occult residual disease represents the primary driver of mortality (15, 16). In this compromised surgical environment, our model demonstrates that adjuvant chemotherapy provides a statistically significant survival rescue effect (P = 0.048), serving as a necessary compensatory mechanism (19–21). In contrast, when a quality-controlled, extensive lymphadenectomy (≥ 21 nodes) is achieved, the surgeon has effectively eliminated the macroscopic disease and regional minimal residual disease (MRD) burden. In this optimized anatomical state, the survival trajectories of patients who received chemotherapy and those who were managed with surgery alone become completely equivalent (P = 0.085), even after rigorous matching for pathological factors (26, 27). This empirical evidence indicates that giving chemotherapy to patients who have already been cured by extensive surgery yields no additional survival benefit, while unnecessarily exposing them to severe systemic hematological and gastrointestinal toxicities (28). Therefore, achieving an upfront clearance of ≥ 21 nodes provides a reliable anatomical gateway to safely exempt patients from chemotherapy, fulfilling the core mandate of precision de-escalation oncology.

A major methodological strength of this study lies in the internal consistency of our predictive architecture. Traditional prognostic nomograms often use parallel Cox models in which chemotherapy is treated as an independent factor, forcing a risk penalty on surgery-only patients regardless of their surgical context. This creates a severe logical contradiction when the stratified data prove chemotherapy is ineffective in certain subcohorts. By deliberately integrating a product interaction term (LND group × adjuvant chemotherapy), we successfully resolved this mathematical conflict. This integration allows the nomogram axes to dynamically adapt: the chemotherapy risk points scale down to nearly zero automatically when the user selects an adequate LND configuration. This architectural refinement ensures that our main predictive model aligns with our layer-specific Kaplan-Meier conclusions. Combined with reliable discrimination in external validation (e.g., 1-year AUC of 0.728, 3-year AUC of 0.663) and good calibration, this interactive system provides surgical oncologists with a reliable, mathematically sound tool for real-world clinical decision-making.

Regarding the anatomical correlate of the 21-node threshold, the relationship between node-count strata and conventional dissection fields deserves explicit consideration. Standardized lymphadenectomy fields in ESCC surgery are conventionally defined by the Japanese Esophageal Society classification, in which two-field dissection encompasses the mediastinal and abdominal compartments, whereas three-field dissection adds the cervical compartment (13). Because neither the SEER registry nor our institutional database records the anatomical extent of the dissection, our node-count threshold cannot be equated with a specific field template on a one-to-one basis. Nevertheless, surgical experience from high-volume centers indicates that a complete, standardized two-field lymphadenectomy in ESCC typically yields approximately 20–30 nodes, whereas yields below this range usually reflect limited or non-standardized resections and yields substantially above it typically require cervical (three-field) extension. On this basis, suboptimal LND (< 21 nodes) in our study should not be equated with a complete two-field dissection but is more consistent with limited or incomplete two-field resections, whereas adequate LND (≥ 21 nodes) is best interpreted as a surrogate for a standardized, complete two-field lymphadenectomy, with three-field procedures representing its upper extension. We acknowledge that the node count is a pathological outcome variable that conflates surgical extent with pathological diligence, and we agree that future prospective studies should record field templates explicitly to verify whether anatomically defined two-field and three-field dissections reproduce the prognostic stratification observed here.

Second, we address the methodological heterogeneity in lymph node dissection between the two cohorts. Propensity score matching balanced the measured baseline covariates (pT, pN, age, and sex) within each surgical stratum, but it cannot balance unmeasured factors such as the surgeon’s field template, the extent of recurrent laryngeal nerve chain dissection, or the intensity of the pathological grossing protocol. The nearly mirror-image distributions of surgical quality between the SEER cohort (89.4% suboptimal) and the Chinese cohort (83.2% adequate) are therefore unlikely to represent patient-level confounding and is more plausibly attributable to systematic differences in operative technique and pathological processing between the two healthcare systems. Two considerations mitigate this concern. First, our primary conclusions rest on within-stratum, within-cohort comparisons of chemotherapy versus surgery alone rather than on direct cross-cohort pooling of survival, so the distributional imbalance does not confound the treatment-effect estimates. Second, the 21-node threshold and the LND-by-chemotherapy interaction were derived exclusively in the SEER cohort and then reproduced in the Chinese cohort, where the nomogram retained acceptable discrimination and calibration despite the diametrically opposed surgical-quality distribution; a threshold that demonstrates prognostic value in two such populations is more likely to reflect a biological constant of regional disease clearance than a local surgical artifact. Nonetheless, we agree that this heterogeneity remains a genuine limitation, and multicenter prospective studies with protocol-defined lymphadenectomy fields and centralized pathological review are required before the threshold can be considered universally applicable.

Third, the pathologically node-positive (pN+) population is biologically heterogeneous and should be stratified into limited nodal disease (pN1) and extended nodal disease (pN2/pN3), with further stratification based on the presence of extracapsular nodal extension. The therapeutic equivalence we observed in the adequate-LND stratum should therefore not be assumed to hold uniformly across these subgroups. Biologically, the capacity of an extended lymphadenectomy to achieve complete regional clearance is plausibly greatest for a limited number of intracapsular deposits (pN1), whereas pN2/pN3 disease, particularly when accompanied by extracapsular extension, implies direct invasion of perinodal tissue and a higher probability of systemic micrometastatic dissemination that cannot be eliminated by local surgical clearance alone. Under this hypothesis, adjuvant chemotherapy may retain a survival benefit for pN2/pN3 patients even after optimal LND, and the adequate-LND stratum in our study is likely enriched for limited pN1 disease, which may partly explain the absence of a chemotherapy effect within it. In the present dataset, formal pN-stratified interaction testing within the adequate-LND stratum is limited by the small number of pN2/pN3 events, and the SEER registry does not record extracapsular extension. We have therefore identified pN-stratified and extracapsular-extension-stratified analyses as priorities for future work: adequately powered studies are needed to determine whether the chemotherapy-exemption strategy proposed here can be extended to patients with extended nodal disease or should be restricted to those with limited, intracapsular pN1 involvement.

Although our interactive model demonstrates robust predictive value, several limitations must be considered. As a retrospective analysis, it is subject to historical practice shifts across the decades spanned by the SEER registry. Additionally, while our single-center Chinese cohort provided a large pool of high-quality surgical cases (≥ 21 nodes, 83.2%), further multicenter prospective trials are warranted to validate these thresholds on a wider scale (29, 30). Furthermore, heterogeneity in surgical field templates and pathological processing between the Western and Eastern cohorts cannot be fully eliminated by propensity score matching, and the adequate-LND stratum may be enriched for limited (pN1) nodal disease; pN-stratified validation, incorporating extracapsular extension where available, is warranted before the chemotherapy-exemption strategy is applied to patients with extended nodal disease.

## 5 Conclusions

In conclusion, this cross-population study demonstrates that a lymph node dissection threshold of 21 nodes is a critical determinant of post-esophagectomy survival in ESCC. More importantly, it acts as a decisive biomarker for tailoring systemic management. Patients with fewer than 21 nodes removed require adjuvant chemotherapy to counter inadequate surgical clearance (P = 0.048), whereas those with a quality-controlled clearance of 21 nodes or more gain no additional survival benefit from cytotoxic regimens and can be safely exempted (P = 0.085), thereby achieving personalized therapeutic de-escalation and preventing toxic overtreatment.

## Data Availability

The data analyzed in this study is subject to the following licenses/restrictions: Publicly Available Dataset: The training cohort data utilized in this study was derived from the Surveillance, Epidemiology, and End Results (SEER) database, which is a publicly available repository. Qualified researchers can access these de-identified data by requesting permission directly from the SEER program. Restricted Local Dataset: The validation cohort data consists of institutional real-world records from Fujian Medical University Union Hospital. Due to institutional regulations and patient privacy protection policies, this local dataset is not publicly available. However, anonymized data extracts can be made available by the corresponding author upon reasonable academic request, subject to approval by the hospital’s review board. Requests to access these datasets should be directed to Yicheng Huang, hyc20010603@163.com.
